# Downregulation of exosomal *miR-7-5p* promotes breast cancer migration and invasion by targeting *RYK* and participating in the atypical WNT signalling pathway

**DOI:** 10.1186/s11658-022-00393-x

**Published:** 2022-10-09

**Authors:** Zhaoyi Liang, Lu Liu, Ruixia Gao, Chengchuan Che, Ge Yang

**Affiliations:** College of Life Sciences, Qufu Normal University, Qufu, 273165 Shandong China

**Keywords:** Breast cancer, Exosome, *miR-7-5p*, *RYK*, EMT, Atypical WNT pathway

## Abstract

**Background:**

Current studies show that exosomal miRNAs become an important factor in cancer metastasis. Among the many miRNA studies, *miR-7-5p* has not been thoroughly investigated in breast cancer metastasis.

**Methods:**

Bioinformatic screening was performed using extant data from the GEO database, and *miR-7-5p* expression levels in breast cancer cell lines and exosomes were further examined using real-time quantitative PCR (qRT-PCR). The extracted exosomes were characterised by transmission electron microscopy (TEM), particle size analysis and marker protein determination. Cell migration and invasion were then examined using wound healing assays and Transwell assays, respectively. Correlation between *miR-7-5p* and receptor-like tyrosine kinase (*RYK*) was analysed by luciferase reporter. The effect of *miR-7-5p* against *RYK*-related downstream factors was verified using western blot assays.

**Results:**

In this study, we found that the expression of *miR-7-5p* was significantly different in exosomes secreted from breast cancer cell lines with different high and low invasiveness. Further experiments revealed that *miR-7-5p* has an important role in inhibiting the migration and invasion of breast cancer. In terms of mechanism of action, *miR-7-5p* was found to target the *RYK*, leading to its reduced expression, which in turn caused a reduction in the phosphorylation level of the downstream factor JNK. Reduced levels of phosphorylated JNK factors lead to reduced levels of phosphorylation of c-Jun protein, which in turn leads to increased expression of EMT transcription factors, thereby inhibiting the epithelial–mesenchymal transition (EMT) process to suppress the invasion of breast cancer.

**Conclusion:**

Thus, we demonstrated that exosomes loaded with high levels of *miR-7-5p* emitted from less aggressive breast cancers can participate in the atypical WNT pathway by targeting the *RYK* gene and thus inhibit breast cancer metastasis.

**Supplementary Information:**

The online version contains supplementary material available at 10.1186/s11658-022-00393-x.

## Introduction

In recent years, breast cancer has been increasing in incidence each year and has become the number one killer threatening women’s health [1, 2]. Breast cancer often results in death due to the uncontrollable spread of tumour cells, causing a decline, or even failure, in the function of other organs [3, 4]. Despite the current advances in the treatment of primary breast cancer, there is as yet no effective treatment for metastatic breast cancer. Since 90% of breast cancer-related deaths are caused by metastatic breast cancer, a clearer insight into the mechanisms of breast cancer metastasis is important for the development of effective treatments for metastatic breast cancer [5].

Exosomes are extracellular vesicles approximately 40–120 nm diameter that are separated from cells and are present in body fluids (serum, urine, etc.) [6, 7]. The exosome was thought to function primarily as an intracellular waste remover when it was first discovered [8], but as research has continued, more and more studies have revealed that its function is not so simple. Exosomes can be loaded with a large number of biologically active molecules (small non-coding RNA, mRNA, DNA, proteins, etc.) released by cells into body fluids, accompanying the flow of body fluids and binding to target cells membranes to release their contents into the target cells, acting as intercellular information transfer [8–10]. MicroRNAs (miRNAs) are non-coding single-stranded RNA molecules of up to 22 nucleotides, which are essential components of the exosome. It induces protein translation inhibition in an unknown manner, primarily by complementary binding to the 3′ non-coding region (3′ UTR) portion of the target mRNA target, which in turn inhibits protein synthesis and regulates intracellular processes by modulating the translation of a set of key mRNAs [11, 12]. Since the first miRNA was discovered in 1993, a steady stream of studies has shown that human miRNAs are instrumental in a variety of cancers [13]. Exosomal miRNAs also reflect to some extent the expression pattern of dysregulated miRNAs in cancer cells. Among these, those miRNAs that are delivered by exosome loading have also been found to be up or down regulated in expression in different tumours, involved in various regulatory mechanisms of cancer growth, apoptosis and metastasis [14, 15].

In the GSE114329 dataset, by analysing the differentially expressed miRNAs of MDA231 EXO and MCF7 EXO, we found that *hsa-miR-7-5p* was highly expressed in exosomes secreted by low-invasive breast cancer cells. Notably, *hsa-miR-7-5p* has been shown to be a potential oncogenic factor involved in regulating the biology of many cancer development processes, including colorectal [16], liver [17, 18], gastric [19] and lung cancers [20]. It has also been found in some studies to have a role in drug resistance in breast cancer [21, 22]. However, research on the role and molecular mechanisms of *hsa-miR-7-5p* in breast cancer metastasis is limited. Therefore, we chose to further investigate the specific molecular mechanisms of *hsa-miR-7-5p* in breast cancer metastasis. We validated a novel target of *hsa-miR-7-5p*, *RYK*, and demonstrated the ability to further influence the JNK-mediated atypical WNT signalling pathway. This offers new ideas for the treatment of metastatic breast cancer.

## Materials and methods

### Bioinformatics analysis

The GSE114329 dataset was searched in the GEO database, and two subsets of the MDA231 EXO and MCF7 EXO were selected for bioinformatic analysis. After downloading the raw data from the dataset, the raw data were filtered to remove splicing and decontamination to obtain clean reads which are compared with the reference genome for subsequent information analysis. Bioinformatics analysis was carried out by Shandong Unhelix Biotechnology Corporation.

### Cell culture

The two human breast cancer cell lines used in this study, MDA-MB-231 (TCHu227) and MCF7 (TCHu74), were purchased from Dalian Meilun Biotechnology Corporation. These cells were assayed for mycoplasma, and no mycoplasma contamination was found in any of them. Both cells were cultured in high-sugar DMEM medium (Solarbio, Beijing, China) comprising 10% fetal bovine serum (Biological Industries, Kibbutz Hulda, Israel) in a cell culture incubator at 5% CO_2_ humidity and 37 °C. To obtain cancer-derived exosomes unaffected by fetal bovine serum exosomes, cells were also cultured in high-sugar DMEM medium containing 10% exosome-free fetal bovine serum (System Biosciences, CA, USA). The cells were cultured to the logarithmic stage of growth for the experiment.

### Extraction of exosomes

The cell culture supernatant was collected during the cell culture process, and the cell culture supernatant was centrifuged (Sigma, Germany) at high speed: 300*g* at 4 °C for 10 min, the supernatant was continued at 2000*g* at 4 °C for 15 min and then the supernatant was centrifuged at 10,000*g* at 4 °C for 30 min. Ultracentrifugation (Beckman Coulter, USA) was then performed: the supernatant was centrifuged at 110,000*g* for 90 min at 4 °C, then the supernatant was discarded and the precipitate was resuspended in pre-cooled PBS and continued to be centrifuged at 110,000*g* for 90 min at 4 °C. The precipitate was resuspended in appropriate PBS and stored in a −80 °C refrigerator.

### Transmission electron microscopy

The extracted exosomes were observed for their morphological characteristics by transmission electron microscopy (TEM). The exosome suspension was suspended as droplets on a copper grid for 15 min, then the droplets were taken up by filter paper, followed by a drop of 4% phosphotungstic acid stain for 2 min. Afterwards, the staining solution was blotted away with filter paper. The copper grids were allowed to dry overnight and then observed using TEM (JEOL, Japan).

### Nanoparticle size and zeta potential analyser

The granulometry of the extracted exosomes was analysed using a nanoparticle size and zeta potential analyser (Malvern Panalytical, UK). The exosome suspension was diluted and placed in a quartz cuvette to measure the particle size.

### Cellular uptake assay

Cells were inoculated in six-well plates at 2 mL per well (5 × 10^5^ cells/mL) and incubated for 24 h. Exosomes were labelled using Dio stain (Coolaber, Beijing, China). Following the instructions, the Dio staining solution was first diluted and then mixed with the exosome suspension and incubated at 37 °C for 30 min protected from light. To remove excess dye, the stained exosomes were centrifuged at 110,000*g* for 90 min at 4 °C. The precipitate was then resuspended in PBS and co-incubated with cells for 3 h, and cellular uptake was observed using two-photon confocal microscope (LSM 880 NLO, Germany).

### Wound healing experiment

The digested cells were inoculated in six-well plates (1 × 10^6^ cells per well) and incubated until the cells had grown to about 80% in each well, then three monolayers of cells were scraped at medium distances in each well with the tip of a 10 μL gun. These were washed in PBS twice, and new cell culture medium was added. Then the width of the scratch was measured with an inverted microscope (Olympus, Japan). The cells were treated accordingly and incubated for a further 48 h and the width of the scratch was measured again. Cell migration was assessed by wound healing rate: wound healing rate = (0 h scratch width − 24 h scratch width)/0 h scratch width × 100%.

### Transwell

Transwell chambers with an 8-μm-pore-size polycarbonate membrane at the bottom were used for detecting the migratory and invasive capacity of the cells. Transfected cells (2 × 10^5^) were resuspended in serum-free DMEM medium and added to the upper chamber (matrix gel coated or uncoated), and then 750 μL of DMEM culture medium containing 10% serum was added to the lower chamber. After 24 h incubation at 37 °C in 5% CO_2_, the cells were fixed in 4% paraformaldehyde (Meilunbio, Dalian, China) for 2 min and then in methanol for 20 min. Finally, the cells were stained with 0.1% crystalline violet stain for 15 min, and then the non-migrated cells and the stromal gel coating were gently scraped off the interior of the chambers with a cotton swab and placed on a slide for imaging and counting of the migrated cells under a microscope.

### Cell transfection

During cell transfection, all oligonucleotides and vectors were synthesised by GenePharma (Shanghai, China), including *RYK* small interfering RNA (si*RYK*) and its control (siNC), *miR-7-5p* mimic and inhibitor or its control (miR-NC mimic and miR-NC inhibitor). Cells were transfected using Lipofectamine 3000 (Mei5bio, Beijing, China) or LipoRNAiMAX (Mei5bio) according to the manufacturer’s requirements. Transfections were assayed after 48 h of normal culture. The sequences of miRNA mimics and inhibitors are shown in Additional file 1: Table S1.

### RNA extraction and qRT-PCR

RNAiso (Takara Bio, Japan) for small RNA and RNAiso (Takara Bio, Japan) for plus were used to extract miRNA and total RNA from cells and exosomes. miRNA 1st Strand cDNA Synthesis Kit (Vazyme Biotech, Nanjing, China) and HiScript II 1st Strand cDNA Synthesis Kit (Vazyme Biotech) were used to perform reverse transcription of miRNA and mRNA. RNA levels were quantified using SYBR Green stain (Vazyme Biotech) on an CFX 96Touch (Bio-Rad, USA) for real-time analysis. Real-time analysis was performed using SYBR Green on an CFX 96Touch according to the following protocol. Protocol for miRNA: 95 °C for 5 min, 40 cycles including 95 °C for 15 s and 60 °C for 20 s, 72 °C for 40 s. Protocol for mRNA: 95 °C for 5 min, 40 cycles including 95 °C for 10 s and 60 °C for 30 s. The expression levels of intracellular and exosomal miRNAs were normalised to U6. The expression levels of intracellular mRNAs were normalised to GADPH. Primer sequences are listed in Additional file 1: Table S2.

### Western blot

Cells or exosomes were first lysed with Western and IP Cell Lysis Solution (Coolaber) and incubated on ice for 15 min, and then the supernatant was gathered by centrifugation. Protein concentration was determined via the BCA protein assay kit (Meilunbio) for quantification. Protein samples were separated on 10% SDS–polyacrylamide gels, protein bands were transferred onto 0.45 µm PVDF membranes (Millipore, Massachusetts, USA). The PVDF films were then sealed with 5% skimmed milk powder (Bio-Rad). The closed PVDF membrane was incubated at 4 °C overnight with the corresponding primary antibody, washed with TBST buffer and incubated with secondary antibody (Proteintech, Wuhan, China) for 4–5 h at 4 °C. Washing with TBST buffer continued at the end of the secondary antibody incubation, followed by observation of the protein bands on the membrane using ECL luminescent solution (Meilunbio) and an imaging system (Bio-Rad). The primary antibodies included: TSG101 (1:20,000, cat. no. 67381-1-Ig), Alix (1:10,000, cat. no. 67715-1-Ig), CD63 (1:10,000, cat. no. 67605-1-Ig), RYK (1:1000, cat. no. 22138-1-AP), E-cadherin (1:5000, cat. no. 20874-1-AP), N-cadherin (1:6000, cat. no. 66219-1-Ig), ZEB1 (1:1000, cat. no. 21544-1-AP), Phospho-JNK (1:2000, cat. no. 80024-1-RR), Phospho-c-Jun (1:2000, cat. no. 28891-1-AP) and β-actin (1:10,000, cat. no. 66009-1-Ig) were obtained from Proteintech.

### Luciferase reporter assay

Using the Biology website (http://www.targetscan.org) to predict the binding site of *miR-7-5p* to *RYK*. The pmirGLO vectors for *RYK*-WT and *RYK*-MUT were then constructed (GenePharma). Logarithmic growth phase MDA-MB-231 cells were inoculated onto 12-well plates at a density of 5 × 10^5^ cells per well. The miR-NC mimics or miR-7-5p mimics, RYK-WT and RYK-MUT, were transfected into MDA-MB-231 cells when cell confluency reached 70%. The reporter plasmid content was 2 µg per well, while miR-7-5p mimics or miR-NC were transfected at a total of 40 pmol per well. After 48 h of transfection, cell lysis was performed and luciferase activity was measured by the Luciferase Assay Kit (Meilunbio).

### Statistical analysis

Data are presented as mean ± standard deviation (SD) (*n* = 3). For comparisons between the two groups, Student’s *t*-test was used to determine the difference between the means of the normal distribution. Significance analysis was performed on all data using GraphPad Prism 5 (San Diego, CA, USA) software, and probability levels of < 0.05 were considered significant.

## Results

### Exosomes from highly invasive breast cancers are more likely to promote cancer cell migration than less invasive ones

**Fig. 1:**
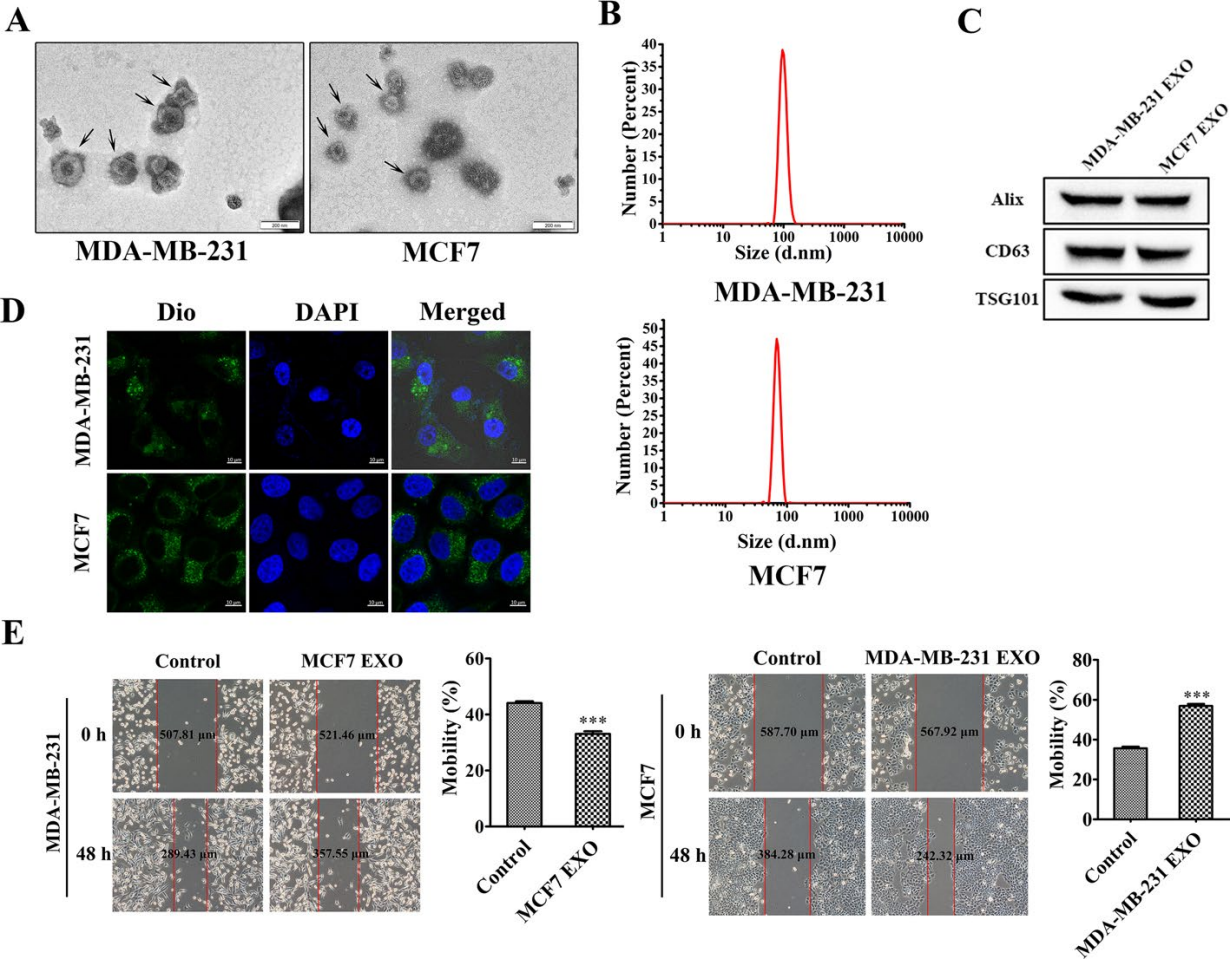
Exosomes from highly invasive breast cancers are more likely to promote cancer cell migration than less invasive ones. **A** Transmission electron microscopy of exosomes extracted from MDA-MB-231 and MCF7 breast cancer cell lines. Scale bar, 200 nm. **B** Particle size distribution of isolated exosomes. **C** Western blot analysis of Alix, TSG101 and CD63, marker proteins of exosomes isolated from MDA-MB-231 and MCF7 cells. **D** Exosomes were labelled using Dio stain and co-incubated with MCF7 and MDA-MB-231 cells for 3 h. Scale bar, 10 μm. **E** Wound healing experiments with MCF7 and MDA-MB-231 cells treated with MDA-MB-231 exosomes and MCF7 exosomes, respectively. Data are presented as mean ± SD (*n* = 3). *p < 0.05, **p < 0.01, ***p < 0.001

The finding that cancer cells secrete more exosomes than normal cells further suggests that exosomes secreted by cancer cells may play a central role in their metastatic mechanism. To investigate the role of breast cancer-derived exosomes in breast cancer migration and invasion, exosomes first need to be isolated. We cultured two breast cancer cell lines, MDA-MB-231 cells and MCF7 cells, using a medium containing fetal bovine serum without exosomes. After collecting the cell cultures and extracting the exosomes using differential centrifugation, they were observed using transmission electron microscopy. The classical morphology of exosomes in a homogeneous cup-like structure was observed as shown in Fig. 1A. We examined the particle size of the extracted material using a nanoparticle size and zeta potential analyser and found that most of the particles were around 100 nm in size (Fig. 1B). The presence of these three marker proteins was further demonstrated using antibodies to CD63, TSG101 and Alix (Fig. 1C). All these results indicated that the extracted vesicular structures were indeed exosomes. After isolation of the exosomes, we co-cultured MCF7-derived exosomes with MDA-MB-231 cells and MDA-MB-231-derived exosomes with MCF7 cells. The co-cultured cells were also subjected to a wound healing assay to verify whether there were some functional differences between the two different exosomes. Furthermore, to determine that exosomes from different sources could enter different cells, we first labelled the extracted exosomes with Dio staining and then observed them with a two-photon laser confocal microscope. The results showed that exosomes were able to enter cells of different origins (Fig. 1D). And the results of the wound healing assay also showed that highly invasive breast cancer-derived exosomes promoted cell migration more than less invasive ones (Fig. 1E).

### Bioinformatic analysis to screen for differentially expressed miRNAs

**Fig. 2:**
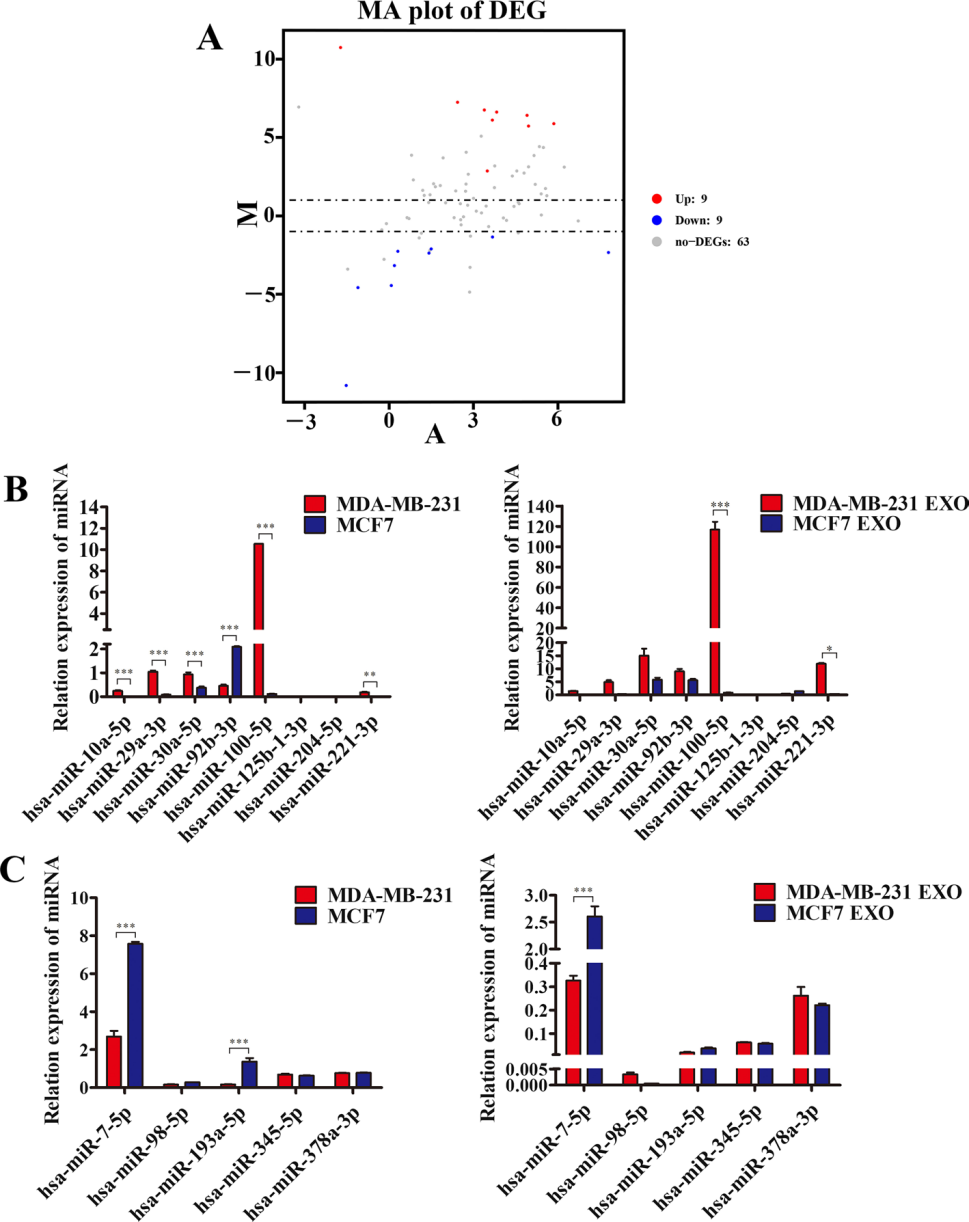
Bioinformatic analysis to screen for differentially expressed miRNAs. **A** Bioinformatic analysis of the volcano plot results showed that MDA-MB-231 and MCF7 source differentially expressed miRNAs in exosomes. **B** The expression of selected eight up-regulated miRNAs in exosomes and cells was examined using real-time quantitative PCR. **C** Real-time quantitative PCR was used to detect the expression of selected five down-regulated miRNAs in exosomes and cells, respectively. Data are presented as mean ± SD (*n* = 3). *p < 0.05, **p < 0.01, ***p < 0.001

It is well known that miRNAs from cancer-derived exosomes are important regulatory molecules that mediate the link between cancer cells and host cells, so we investigated whether differentially expressed miRNAs within exosomes from different sources were responsible for mediating the functional differences between the two. We used the GSE114329 dataset from the GEO database to screen for differentially expressed miRNAs in two subsets of MDA231 EXO and MCF7 EXO by bioinformatics analysis. Nine relatively high and nine relatively low miRNAs were screened in exosomes produced by MDA-MB-231 cells (Fig. 2A). Several miRNAs with large differences in the replicate data were further excluded, several miRNAs that did not show significant differences probably owing to experimental reasons were selected, and finally eight miRNAs that were relatively highly expressed in exosomes produced by MDA-MB-231 cells and five miRNAs that were relatively lowly expressed were selected (Fig. 2A; Additional file 1: Table S3). We used qRT-PCR to validate the expression of several miRNAs in cells and exosomes. The experiments showed that *hsa-miR-7-5p* and *hsa-miR-100-5p* were most clearly different in cells and exosomes (Fig. 2B, C). We first selected *hsa-miR-7-5p* as our study subject. It was clear that *hsa-miR-7-5p* was significantly more expressed in low-invasive cells and exosomes than in highly invasive cells and exosomes.

### Downregulation of exosomal miR-7-5p expression significantly facilitates the migration and invasion of breast cancer cells

**Fig. 3:**
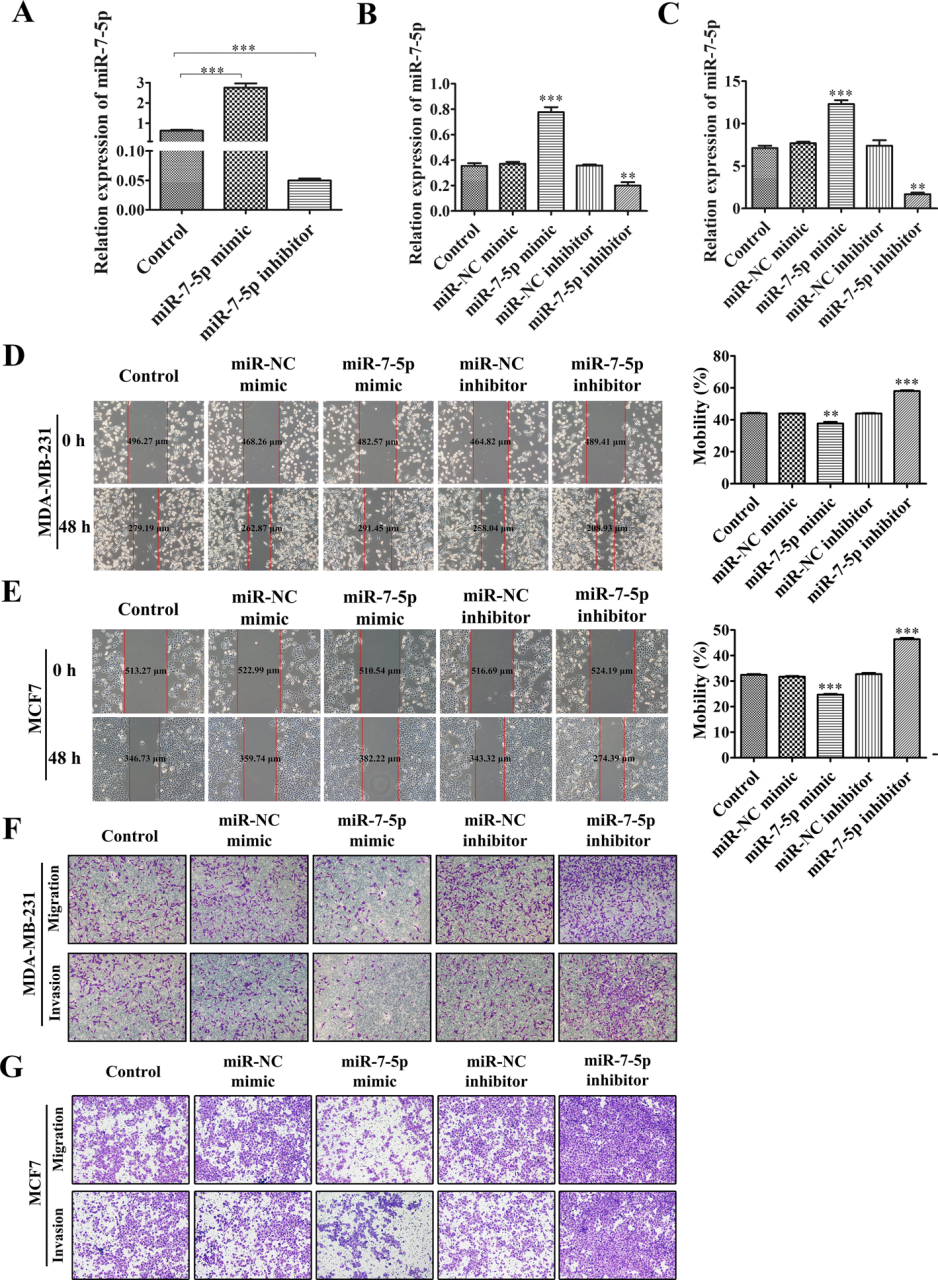
Downregulation of exosomal *miR-7-5p* expression significantly facilitates the migration and invasion of breast cancer cells. **A** Expression of *miR-7-5p* in the secreted exosomes of blank control, *miR-7-5p* mimic and suppressor transfected MDA-MB-231 cells was measured using qRT-PCR assay. **B**, **C** The expression of *miR-7-5p* in MDA-MB-231 cells and MCF7 cells after transfection of blank control, *miR-7-5p* mimics and inhibitors and their negative controls was measured using qRT-PCR assay. **D**, **E** Wound healing assay using blank control, *miR-7-5p* mimic and inhibitor and its negative control after treatment of MDA-MB-231 cells and MCF7 cells. **F**, **G** The migratory invasion ability of cells after treatment of MDA-MB-231 cells and MCF7 cells with blank control, *miR-7-5p* mimics and inhibitors and their negative control was examined using Transwell assay. Data are presented as mean ± SD (*n* = 3). *p < 0.05, **p < 0.01, ***p < 0.001

To further determine whether microRNAs function in dependence on exosomes for delivery, we used transfection experiments to increase or decrease intracellular expression of *miR-7-5p*. Cell cultures of transfected cells were then collected, and the exosomes released from the transfected cells were isolated. Whether changes in intracellular *miR-7-5p* levels cause corresponding changes in *miR-7-5p* within the exosomes was investigated by qRT-PCR assay. The experiments conclusively demonstrated that an increase in intracellular *miR-7-5p* also increased the amount of *miR-7-5p* in the exosomes they released, and similarly, a decrease in *miR-7-5p* expression led to a decrease in *miR-7-5p* expression in the exosomes the cells released (Fig. 3A). This suggests that intracellular *miR-7-5p* acts through exosomal delivery, at least to some extent.

We next investigate what role *miR-7-5p* plays in the intercellular delivery of breast cancer. We used wound healing assays and Transwell assays to investigate whether *miR-7-5p* has an effect on the migration invasion of breast cancer cells. We first verified that both mimics and inhibitors had been transfected into the cells within 48 h by qRT-PCR (Fig. 3B, C). The results of the wound healing and Transwell assays showed that the migration invasion ability of breast cancer cells in the *miR-7-5p* mimic group was significantly reduced, while the migration invasion ability of breast cancer cells in the *miR-7-5p* inhibitor group was significantly increased (Fig. 3D–G). This demonstrated that *miR-7-5p* significantly depressed breast cancer migration and invasion, which corresponds to our previous demonstration that *miR-7-5p* is highly expressed in low-invasive breast cancer cell lines and their exosomes, but lowly expressed in high-invasive breast cancer cell lines and their exosomes.

### *Hsa-miR-7-5p* targets the *RYK* gene

**Fig.4:**
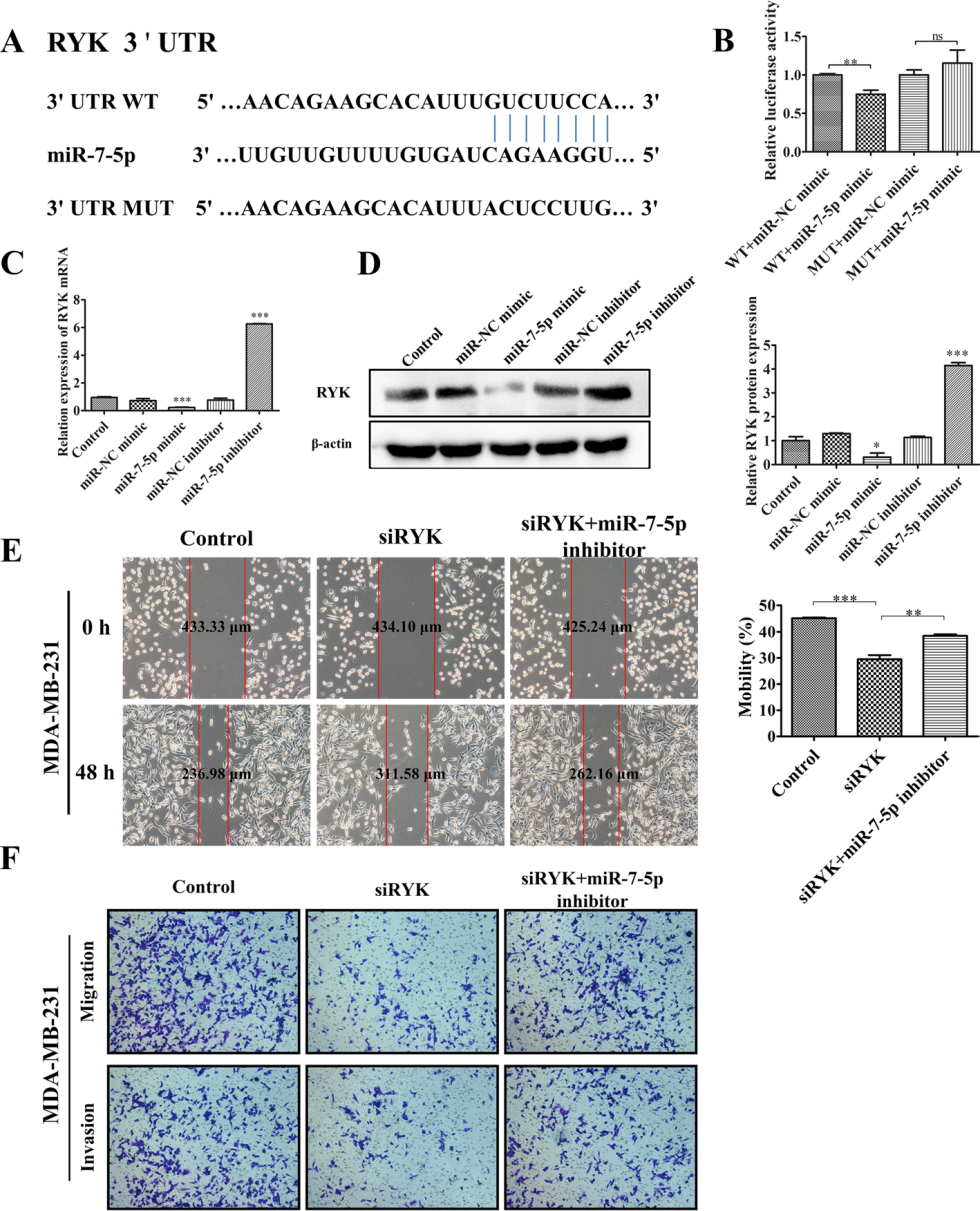
*Hsa*-*miR-7-5p* targets the *RYK* gene. **A** The wild type (WT) and mutant (MUT) of *miR-7-5p* at the binding site of the 3′ UTR of the *RYK* gene. **B** Validation of *miR-7-5p* targeting to *RYK* using luciferase reporter gene assays. MDA-MB-231 cells were co-transfected with negative control (NC) or *miR-7-5p* mimics and luciferase reporter plasmids (including *RYK* 3′ UTR WT or *RYK* 3′ UTR MUT). **C** Expression of *RYK* mRNA in MDA-MB-231 cells transfected with NC or *miR-7-5p* mimics and inhibitors was measured by quantitative real-time PCR. **D** Western blot analysis of RYK protein expression in MDA-MB-231 cells transfected with NC or *miR-7-5p* mimics and inhibitors. **E** Wound healing experiments using blank control, si*RYK* and si*RYK* + *miR-7-5p* inhibitor after co-treatment of MDA-MB-231 cells. **F** A Transwell assay was used to verify the migratory invasion ability of MDA-MB-231 cells treated with blank control, si*RYK* and si*RYK* + *miR-7-5p* inhibitor. Data are presented as mean ± SD (*n* = 3). *p < 0.05, **p < 0.01, ***p < 0.001

To further investigate the specific molecular mechanism by which *miR-7-5p* presents a significant inhibitory effect in breast cancer migration invasion, we utilised four miRNA target gene prediction software (miRTarBase, miRDB, TargetScan, miRWalk) to predict candidate target genes for *miR-7-5p*. We screened 41 candidate target genes (Additional file 1: Fig. S1), and then genes associated with tumour cell migration and invasion were included as further screening requirements. We finally identified the *RYK* genes with high scores as possible target genes for *miR-7-5p*. We next used only highly invasive MDA-MB-231 cells as our study subjects. We predicted the binding site for *miR-7-5p* in the 3′ UTR of the *RYK* gene (wild type) and targeted it for mutation (mutant type) and constructed plasmids for transfection (Fig. 4A). The experimental results showed that co-transfected *miR-7-5p* mimics significantly reduced reporter gene expression after the *RYK* wild type 3′ UTR, whereas no change in reporter gene expression was observed in the mutant group (Fig. 4B). Therefore, we concluded that *RYK* is a direct target of *miR-7-5p*. Consistent with the outcome of the dual luciferase reporter gene assay, the *RYK* gene also showed markedly reduced transcript levels and protein expression levels following transfer of *miR-7-5p* mimics (Fig. 4C, D). Our further in vitro experiments showed that *RYK* knockdown significantly inhibited the migratory and invasive ability of MDA-MB-231 cells compared with the control group. This result was identical to that obtained after treatment using *miR-7-5p* mimic. However, the inhibitory effect after *RYK* knockdown was reversed to some extent by *miR-7-5p* inhibitor (Fig. 4E, F). Thus, it is evident that exosomal *miR-7-5p* inhibits the migratory invasion of breast cancer to some extent by targeting the *RYK* gene.

### *Hsa-miR-7-5p* is involved in the JNK-mediated atypical WNT signalling pathway and significantly inhibits breast cancer metastasis

**Fig. 5:**
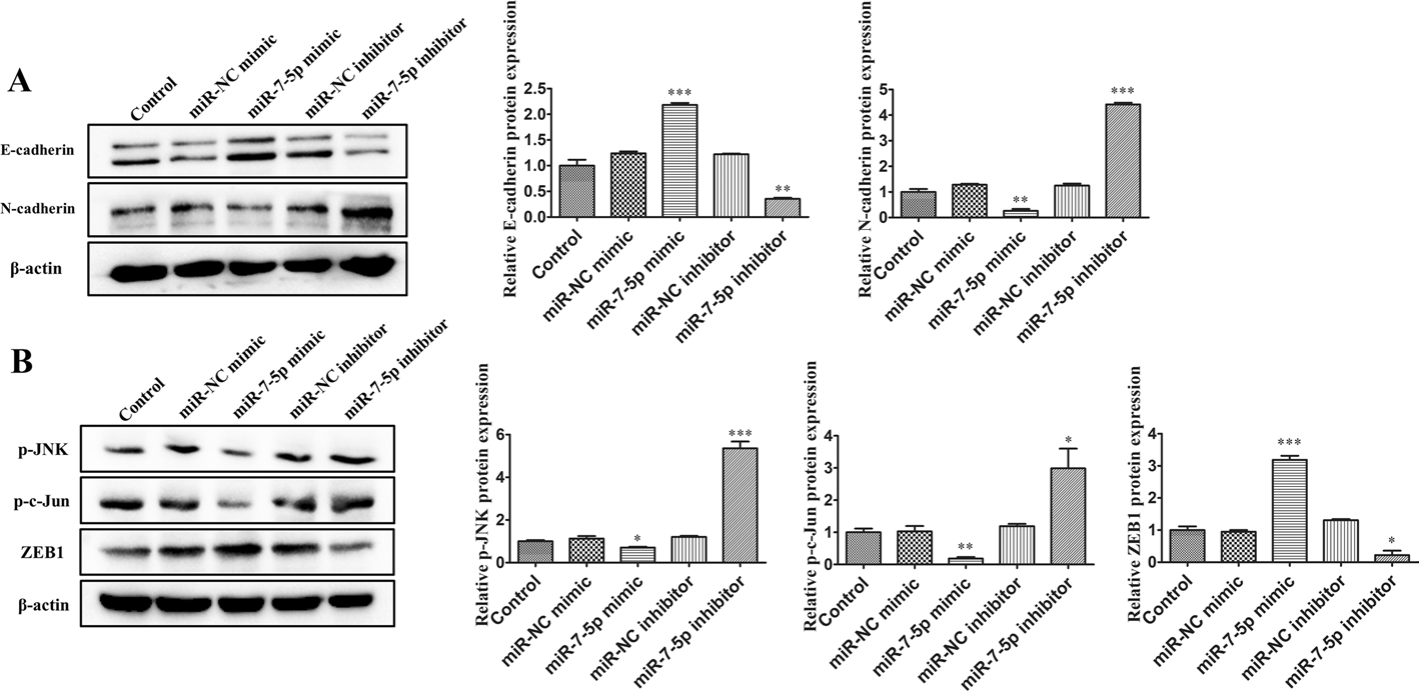
*Hsa-miR-7-5p* is involved in the JNK-mediated atypical WNT signalling pathway and significantly inhibits breast cancer metastasis. **A** Western blot analysis of E-cadherin and N-cadherin protein expression levels after treatment of MDA-MB-231 cells with blank controls or *miR-7-5p* mimics, inhibitors and their negative controls. **B** Western blot analysis of p-JNK, p-c-Jun and ZEB1 protein expression levels after treatment of MDA-MB-231 cells with blank control or *miR-7-5p* mimics, inhibitors and their negative controls. Data are presented as mean ± SD (*n* = 3). *p < 0.05, **p < 0.01, ***p < 0.001

Numerous studies have demonstrated that the migratory and invasion of breast cancer is closely related to the development of EMT. Our experiments also show that transfection of *miR-7-5p* mimics promotes the expression of E-cadherin and inhibits the expression of N-cadherin (Fig. 5A). Thus, *miR-7-5p* can inhibit the development of EMT. It has also been demonstrated that JNK and c-Jun in the atypical WNT signalling pathway, in which *RYK* is involved, can be involved in EMT development [23], which prompted us to investigate whether JNK and c-Jun, which are downstream factors of the RYK receptor, can be affected by *miR-7-5p*. We found that downregulation of *miR-7-5p* in breast cancer cells promoted a cascade response of the atypical WNT pathway, that is, phosphorylation of JNK and c-Jun proteins (Fig. 5B). Phosphorylated c-Jun protein inhibits the expression of the EMT core transcription factor ZEB1 (Fig. 5B), thereby suppressing the expression of E-cadherin and promoting the expression of N-cadherin, thereby facilitating the EMT process and promoting breast cancer metastasis. Ultimately, we demonstrated that *miR-7-5p* can inhibit breast cancer migration invasion by targeting the *RYK* gene, which is involved in the JNK-mediated atypical WNT signalling pathway.

## Discussion

Breast cancer is currently the leading malignancy threatening women’s mortality worldwide [24]. Metastatic malignant breast cancer accounts for 20–30% of breast cancer cases and the survival rate of the disease is only 22%, making it the leading cause of death for patients [5, 25, 26]. Metastatic spread of breast cancer involves many steps, including EMT, migration, invasion, extravasation, mesenchymal–epithelial transformation (MET) and colonisation [27]. An increasing range of research has demonstrated the importance of exosomes for all metastatic processes such as migration invasion of cancer [28, 29]. It has been shown that cancer cells secrete much higher levels of exosomes than those released by normal cells, and therefore the exosomes secreted by cancer cells themselves are critical for the exchange of genetic information and the reprogramming of metabolism between cells, as well as the microenvironment in which the cells live [30, 31]. We screened through validation that *miR-7-5p* was relatively highly expressed in low-invasive breast cancer cell lines and less expressed in highly invasive cells. Further investigation of the mechanism of *miR-7-5p* action revealed that the *RYK* gene may act as a new target gene for *miR-7-5p*.

Among these, the *RYK* gene is a receptor-like tyrosine kinase, characterised by impaired kinase activity, which can exert regulatory effects by binding WNT ligands through its structural region [32]. In fact, *RYK* has been studied extensively in human development and more initially in cancer [33]. However, in recent years, *RYK* has been investigated as a receptor in atypical signalling pathways in many cancers [32, 34]. One study found that inhibition of β-catenin, a part of the typical WNT pathway, did not antagonise the *RYK*-induced metastatic phenotype in gastric cancer, demonstrating that the pro-metastatic effects of *RYK* in gastric cancer are mediated, at least in part, through the atypical Wnt signalling pathway [34]. It has been demonstrated that in prostate cancer the WNT5A/RYK signalling pathway is involved in the apoptotic and proliferative processes of cancer [35]. It has also been demonstrated that, as a response to WNT5A, *RYK* promotes the proliferation of breast stem and progenitor cells, and further studies have found that *RYK* plays a role as a receptor for the atypical WNT signalling pathway in the promotion of brain metastasis in breast cancer [36]. And in glioblastoma, *RYK* knockdown inhibited the invasiveness of wnt5a-induced U-105MG and U251MG glioma cells [37]. Various studies have demonstrated that abnormal WNT/RYK signalling may promote the oncogenic properties of cancer, particularly in tissues where *RYK* development is important. It has been reported that the atypical WNT pathway is often not dependent on β-catenin, but instead involves small GTPases of the Rho family, such as Rho or Rac, and that signalling enhances cell motility through the JNK/c-JUN cascade signalling response [36]. However, phosphorylated c-Jun protein is often able to affect the expression of EMT transcription factors (e.g. ZEB1) and thereby influence the EMT process. EMT is widely known to be an important process affecting the migration and invasion of breast cancer [23, 38, 39]. Interestingly, we demonstrated experimentally that *miR-7-5p* expression was significantly positively correlated with E-cadherin expression and significantly negatively correlated with N-cadherin expression. This indicates that *miR-7-5p* can inhibit the EMT process. We further experimentally verified that *miR-7-5p* was also able to affect the phosphorylation of JNK and c-Jun protein and ZEB1 protein.

Therefore, we determined that *miR-7-5p* targets the *RYK* gene, causing deletion of the RYK receptor and affecting the JNK-mediated cascade of the atypical WNT pathway, leading to effects on the phosphorylation of JNK and c-Jun proteins, resulting in increased expression of EMT transcription factors such as ZEB1. This in turn affects the expression of E-cadherin and suppresses EMT, ultimately inhibiting the migration and invasion of breast cancer (Additional file 1: Fig. S2).

There are of course limitations to our study. (1) We were not able to analyse the clinical significance of exosomal *miR-7-5p* in breast cancer by collecting case samples of tissue. (2) Given the limited experimental conditions, we should have used more breast cancer cell lines to validate our results. (3) We should further validate the effect of *miR-7-5p* in vivo by nude mouse tumorigenesis assay (Additional file 2).

## Conclusions

In conclusion, our experimental data show that *miR-7-5p* in exosomes is closely associated with breast cancer migration and invasion. We identified a new target factor for *miR-7-5p*, *RYK*. We linked the oncogenic effect of *miR-7-5p* to the atypical WNT signalling pathway via the *RYK* gene. Our findings provide new targets for the treatment of advanced breast cancer and new ideas for the study of the molecular mechanisms of miRNAs.

## Supplementary Information

**Additional file 1: Table S1**. Sequences of oligonucleotide fragment. **Table S2** Sequences of primers required for the experiment. **Table S3** Relative expression of several miRNAs screened from the GSE114329 dataset.

**Additional file 2.**

## Data Availability

Please contact the corresponding author for data requests.

## Abbreviations

qRT-PCR: Real-time quantitative PCR
EXO: Exosome
TEM: Transmission electron microscopy
*RYK*: Receptor-like tyrosine kinase
EMT: Epithelial–mesenchymal transition
miRNAs: MicroRNAs
3′ UTR: 3′ non-coding region
WT: Wild type
MUT: Mutant type
MET: Mesenchymal–epithelial transformation
p-JNK: Phosphorylated JNK protein
p-c-Jun: Phosphorylated c-Jun protein
