# Supplementary material for: Downregulation of exosomal miR-7-5p promotes breast cancer migration and invasion by targeting RYK and participating in the atypical WNT signalling pathway
Source: Cell Mol Biol Lett. 2022 Oct 9;27:88. doi: 10.1186/s11658-022-00393-x (PMC9549651; doi:10.1186/s11658-022-00393-x)
Supplement: Supplementary file 1 — Additional file 1: Table S1. Sequences of oligonucleotide fragment. Table S2 Sequences of primers required for the experiment. Table S3 Relative expression of several miRNAs screened from the GSE114329 dataset. [file 11658_2022_393_MOESM1_ESM.docx]

Table 1 Sequences of oligonucleotide fragment

| Oligonucleotides | Sequences (5’-3’) |
| --- | --- |
| Mimics negative control | Sense: UUCUCCGAACGUGUCACGUTT |
|  | Antisense: ACGUGACACGUUCGGAGAATT |
| Has-miR-7-5p mimics | Sense: UGGAAGACUAGUGAUUUUGUUGUU |
|  | Antisense: CAACAAAAUCACUAGUCUUCCAUU |
| Inhibitor negative control | CAGUACUUUUGUGUAGUACAA |
| Has-miR-7-5p inhibitor | AACAACAAAAUCACUAGUCUUCCA |
| SiRYK | Sense: GGUGAAGGAUAUAGCAAUAUC |
|  | Antisense: UAUUGCUAUAUCCUUCACCUU |

Table 2 Sequences of primers required for the experiment

| Genes | Primer sequences (5’-3’) |
| --- | --- |
| RYK | Forward: CTTTATCAGTGTTTCGGGTAG  Reverse: GCGTAGAAGTGGTTGGAGC |
| GADPH | Forward: AGCCACATCGCTCAGACAC  Reverse: GCCCAATACGACCAAATCC |
| U6 | Reverse transcription: TGGTGTCGTGGAGTCG  Forward: CTCGCTTCGGCAGCACA  Reverse: AACGCTTCACGAATTTGCGT |
| Has-miR-7-5p | Reverse transcription:  GTCGTATCCAGTGCAGGGTCCGAGGTATTCGCACTGGATACGACACAACA  Forward: GCGCGTGGAAGACTAGTGATTT  Reverse: AGTGCAGGGTCCGAGGTATT |
| Has-miR-98-5p | Reverse transcription: GTCGTATCCAGTGCAGGGTCCGAGGTATTCGCACTGGATACGACAACAAT  Forward: CGCGCGTGAGGTAGTAAGTTGT  Reverse: AGTGCAGGGTCCGAGGTATT |
| Has-miR-193a-5p | Reverse transcription: GTCGTATCCAGTGCAGGGTCCGAGGTATTCGCACTGGATACGACTCATCT  Forward: TGGGTCTTTGCGGGCG  Reverse: AGTGCAGGGTCCGAGGTATT |
| Has-miR-345-5p | Reverse transcription: GTCGTATCCAGTGCAGGGTCCGAGGTATTCGCACTGGATACGACGAGCCC  Forward: GCGGCTGACTCCTAGTCCA  Reverse: AGTGCAGGGTCCGAGGTATT |
| Has-miR-378a-3p | Reverse transcription: GTCGTATCCAGTGCAGGGTCCGAGGTATTCGCACTGGATACGACGCCTTC  Forward: CGCGACTGGACTTGGAGTCA  Reverse: AGTGCAGGGTCCGAGGTATT |
| Has-miR-10a-5p | Reverse transcription: GTCGTATCCAGTGCAGGGTCCGAGGTATTCGCACTGGATACGACCACAAA  Forward: CGCGTACCCTGTAGATCCGAA  Reverse: AGTGCAGGGTCCGAGGTATT |
| Has-miR-29a-3p | Reverse transcription: GTCGTATCCAGTGCAGGGTCCGAGGTATTCGCACTGGATACGACTAACCG  Forward: CGCGTAGCACCATCTGAAAT  Reverse: AGTGCAGGGTCCGAGGTATT |
| Has-miR-30a-5p | Reverse transcription: GTCGTATCCAGTGCAGGGTCCGAGGTATTCGCACTGGATACGACCTTCCA  Forward: CGCGTGTAAACATCCTCGAC  Reverse: AGTGCAGGGTCCGAGGTATT |
| Has-miR-92b-3p | Reverse transcription: GTCGTATCCAGTGCAGGGTCCGAGGTATTCGCACTGGATACGACGGAGGC  Forward: GCGTATTGCACTCGTCCCG  Reverse: AGTGCAGGGTCCGAGGTATT |
| Has-miR-100-5p | Reverse transcription: GTCGTATCCAGTGCAGGGTCCGAGGTATTCGCACTGGATACGACCACAAG  Forward: GCGAACCCGTAGATCCGAA  Reverse: AGTGCAGGGTCCGAGGTATT |
| Has-miR-125b-1-3p | Reverse transcription: GTCGTATCCAGTGCAGGGTCCGAGGTATTCGCACTGGATACGACAGCTCC  Forward: GCGACGGGTTAGGCTCTTG  Reverse: AGTGCAGGGTCCGAGGTATT |
| Has-miR-204-5p | Reverse transcription: GTCGTATCCAGTGCAGGGTCCGAGGTATTCGCACTGGATACGACAGGCAT  Forward: CGCGTTCCCTTTGTCATCCT  Reverse: AGTGCAGGGTCCGAGGTATT |
| Has-miR-221-3p | Reverse transcription: GTCGTATCCAGTGCAGGGTCCGAGGTATTCGCACTGGATACGACGAAACC  Forward: CGCGAGCTACATTGTCTGCTG  Reverse: AGTGCAGGGTCCGAGGTATT |

Table 3 Relative expression of several miRNAs screened from the GSE114329 dataset

| Name | Relative exosomal miRNA expression | | | | P value |
| --- | --- | --- | --- | --- | --- |
|  | MDA231-1 | MDA231-2 | MCF7-1 | MCF7-2 |  |
| Has-miR-29a-3p | 117.9361019 | 5.756146605 | 0.762361307 | 0.164600627 | 0.000594555^***^ |
| Has-miR-221-3p | 344.0480767 | 30.15124412 | 6.480071113 | 3.785814422 | 0.001036879^**^ |
| Has-miR-125b-5p | 188.2910869 | 10.14178211 | 1.905903269 | 2.139808151 | 0.002121109^**^ |
| Has-miR-193a-5p | 1.762263592 | 0 | 9.148335689 | 6.913226335 | 0.004528176^**^ |
| Has-miR-204-5p | 17.21595971 | 1.096408877 | 0 | 0 | 0.006008539^**^ |
| Has-miR-345-5p | 0.542234951 | 0 | 4.955348498 | 4.279616303 | 0.007604395^**^ |
| Has-miR-7-5p | 743.9463534 | 3.289226632 | 12.57896157 | 5.431820692 | 0.012006597^*^ |
| Has-miR-100-5p | 42.02320874 | 23.57279086 | 5.717709806 | 1.975207524 | 0.01240907^*^ |
| Has-miR-98-5p | 1.762263592 | 0.548204439 | 7.623613074 | 6.0902232 | 0.019039123^*^ |
| Has-miR-378a-3p | 11.79361019 | 1.096408877 | 30.11327164 | 20.57507838 | 0.02390023^*^ |
| Has-miR-30a-5p | 5.693466991 | 3.015124412 | 0.381180654 | 0.987603762 | 0.197736753 |
| Has-miR-92b-3p | 4.880114563 | 4.385635509 | 1.905903269 | 1.316805016 | 0.227792352 |
| Has-miR-10a-5p | 6.100143204 | 5.482044386 | 3.430625883 | 2.139808151 | 0.380960911 |

*p < 0.05, **p < 0.01 and ***p < 0.001


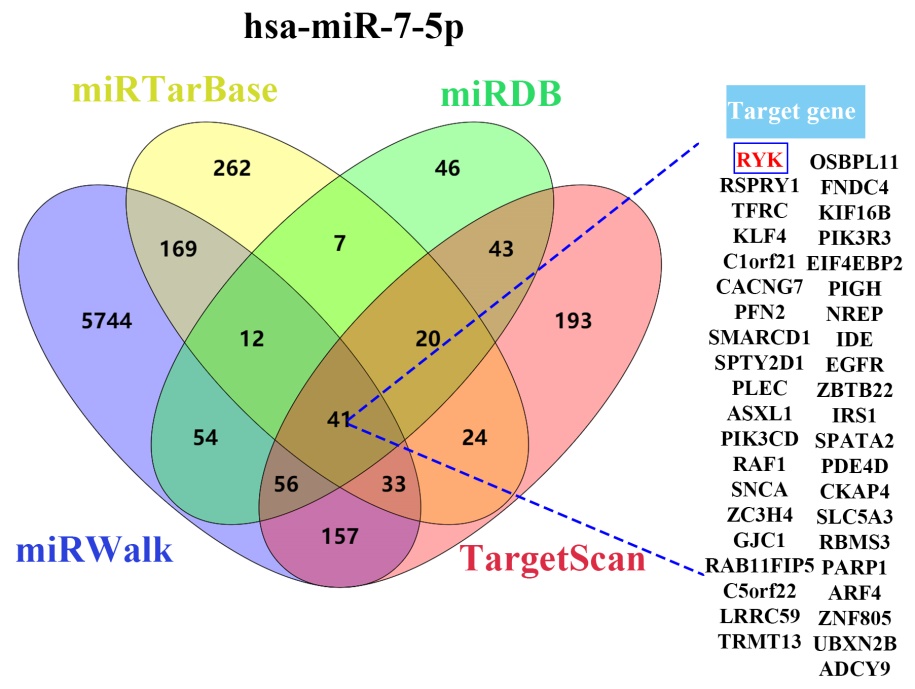


Fig. 1 Four target gene prediction software (miRTarBase, miRDB, TargetScan, miRWalk) were used to predict the target gene of miR-7-5p.


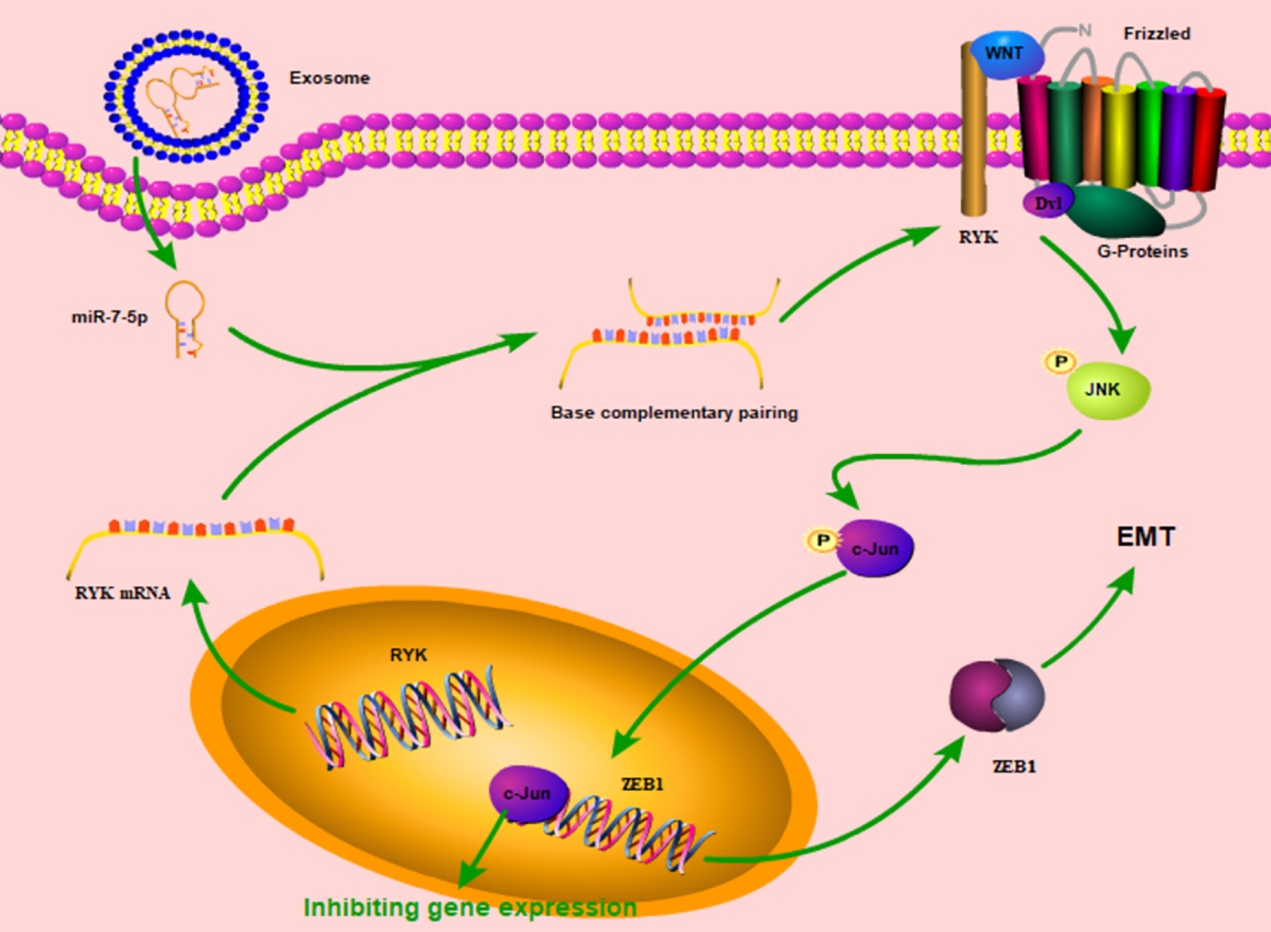


Fig. 2 A model that explicitly demonstrates the effect of exosomal miR-7-5p on breast cancer metastasis. The model illustrates how exosomal delivery of miR-7-5p from breast tumour cells inhibits breast cancer cell metastasis by regulating atypical WNT and EMT-related signalling pathways.
