# Supplementary material for: Downregulation of exosomal miR-7-5p promotes breast cancer migration and invasion by targeting RYK and participating in the atypical WNT signalling pathway
Source: Cell Mol Biol Lett. 2022 Oct 9;27:88. doi: 10.1186/s11658-022-00393-x (PMC9549651; doi:10.1186/s11658-022-00393-x)

Original Western Blot for exosome characterization

Alix

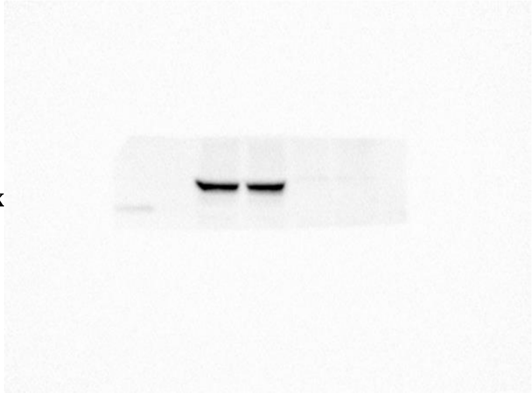

CD63

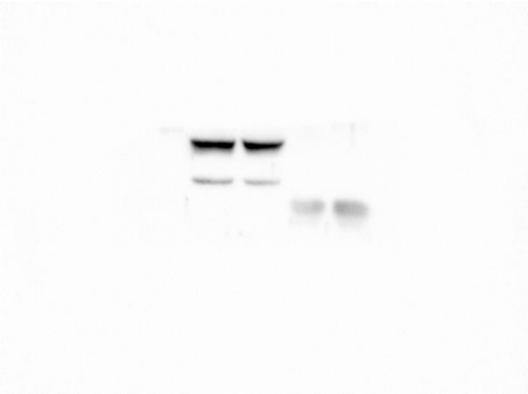

TSG101

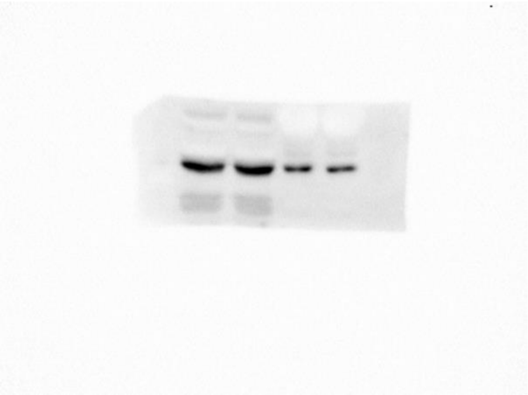

Original Western Blot of RYK expression

RYK

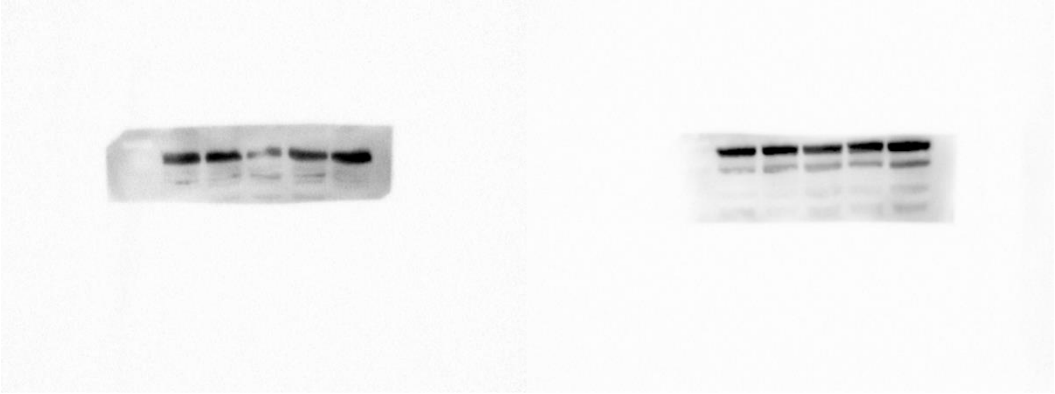

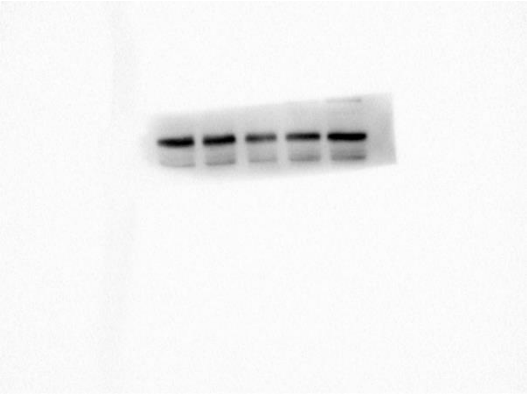

β-actin

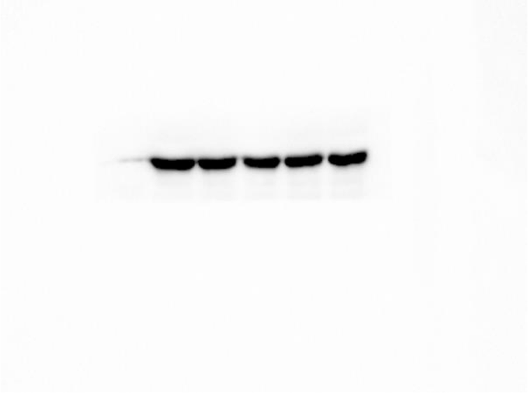

Original Western Blot of EMT-related protein expression

E-cadherin

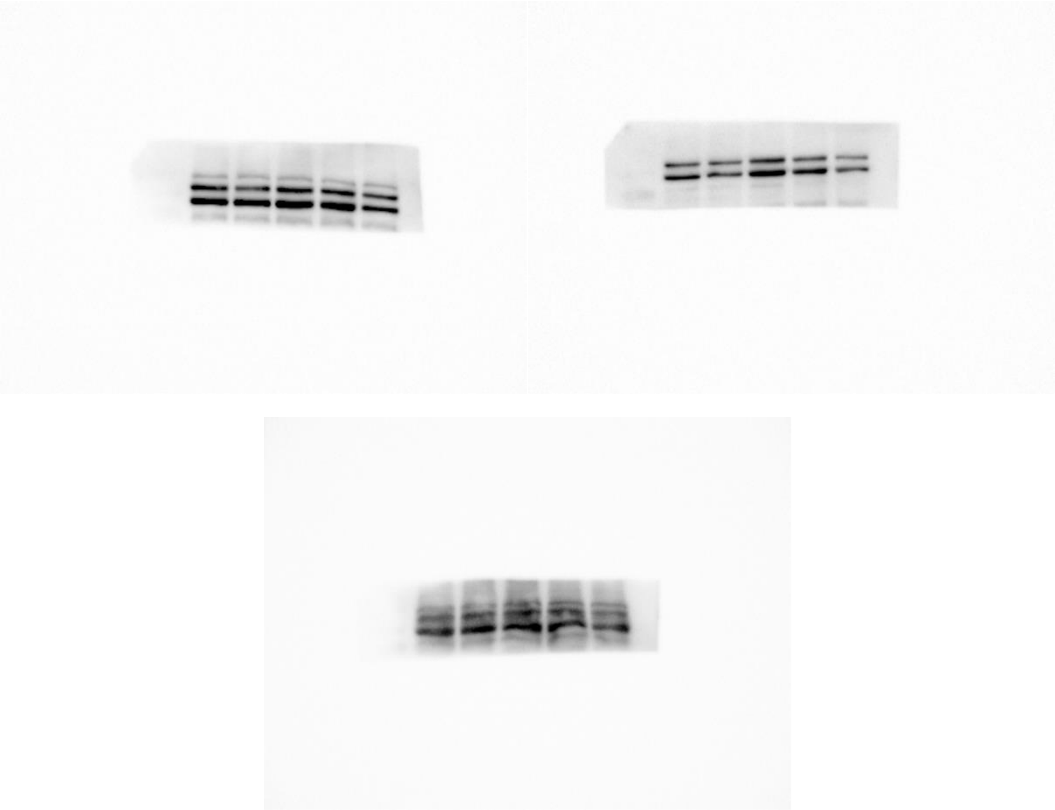

N-cadherin

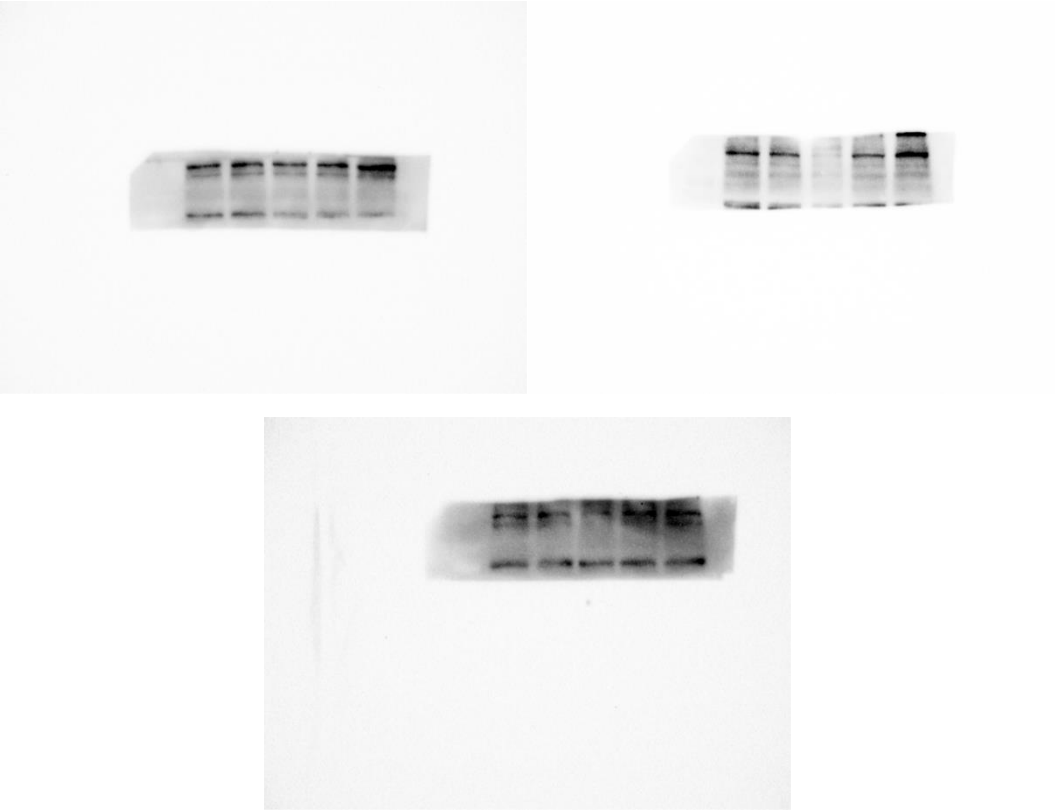

β-actin

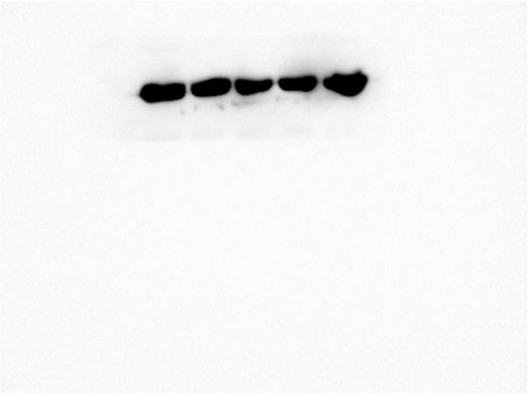

Original Western Blot of pathway-associated protein expression

Phosphorylated JNK（p-JNK）

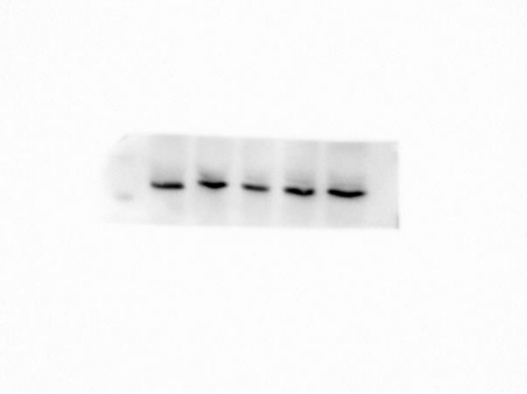

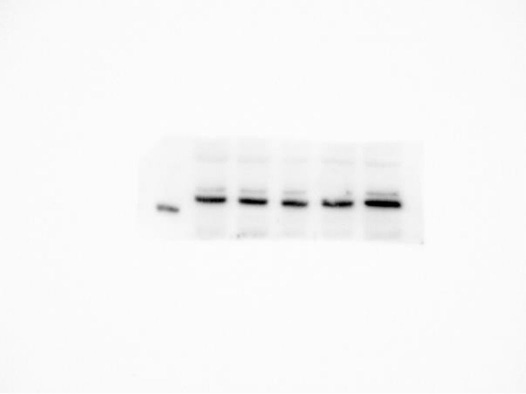

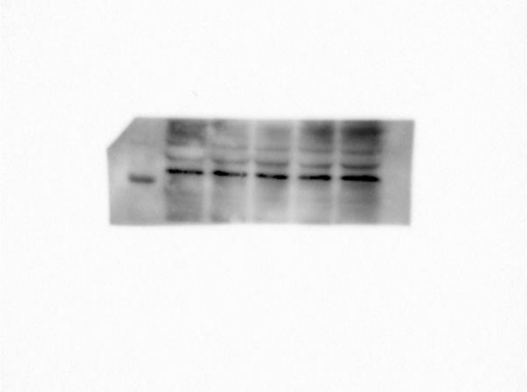

Phosphorylated c-Jun（p-c-Jun）

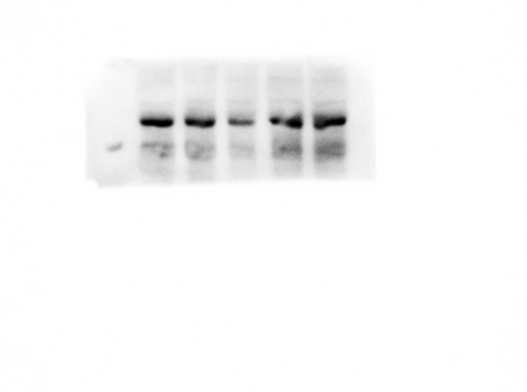

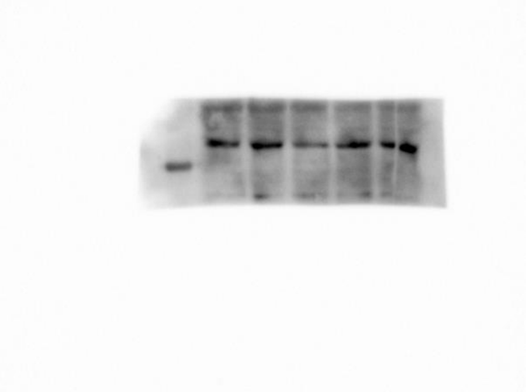

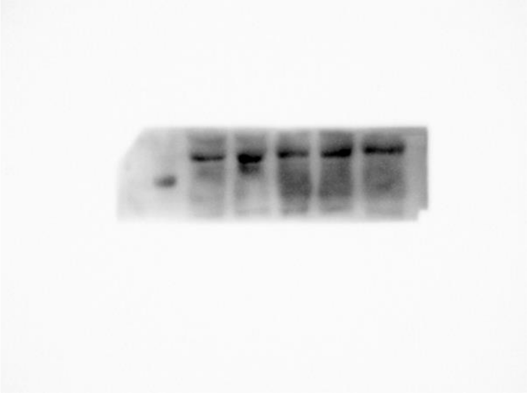

ZEB1

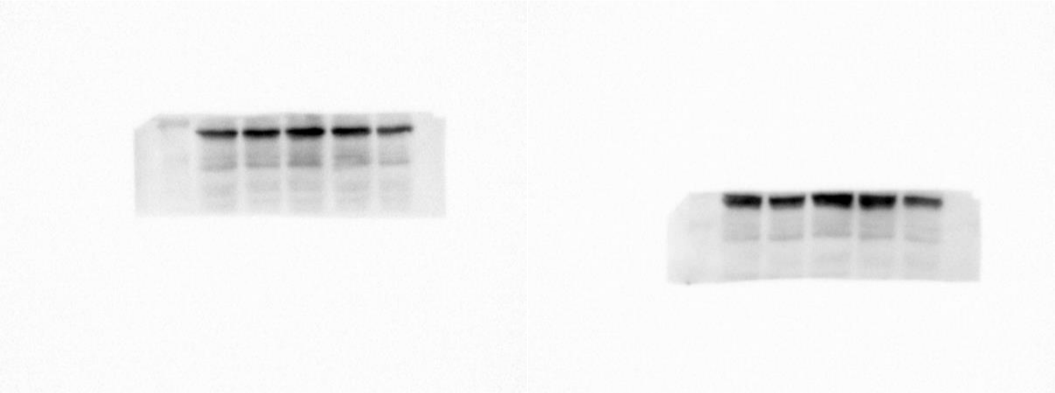

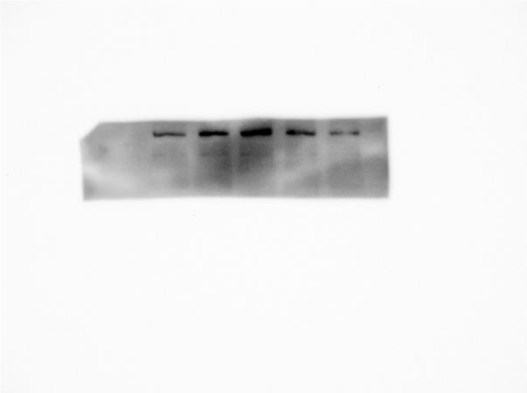

β-actin

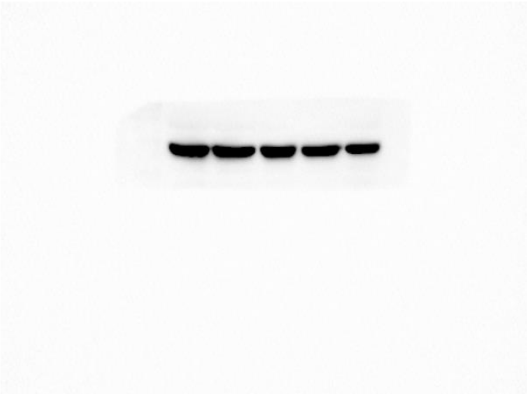

Supplement: Supplementary file 2 — Additional file 2. [file 11658_2022_393_MOESM2_ESM.pdf]
